# Cost-effectiveness of rule-based immunoprophylaxis against respiratory syncytial virus infections in preterm infants

**DOI:** 10.1007/s00431-017-3046-1

**Published:** 2017-11-22

**Authors:** Maarten O. Blanken, Geert W. Frederix, Elisabeth E. Nibbelke, Hendrik Koffijberg, Elisabeth A. M. Sanders, Maroeska M. Rovers, Louis Bont, on behalf of the Dutch RSV Neonatal Network

**Affiliations:** 1 Division of Pediatric Immunology and Infectious Diseases, University Medical Center Utrecht, P.O. Box 85090, 3508 AB Utrecht, the Netherlands; 2 Division Julius Center for Health Sciences and Primary Care, University Medical Center Utrecht, Utrecht, The Netherlands; 3 Department of Health Technology and Services Research, University of Twente, Enschede, The Netherlands; 4 Departments of Epidemiology, Biostatistics and HTA, and Operating Rooms, Radboud University Nijmegen Medical Center, Nijmegen, The Netherlands

**Keywords:** Respiratory syncytial virus, Prophylaxis, Cost-effectiveness analysis, Moderately preterm infants, Prediction rule

## Abstract

The objective of the paper is to assess the cost-effectiveness of targeted respiratory syncytial virus (RSV) prophylaxis based on a validated prediction rule with 1-year time horizon in moderately preterm infants compared to no prophylaxis. Data on health care consumption were derived from a randomised clinical trial on wheeze reduction following RSV prophylaxis and a large birth cohort study on risk prediction of RSV hospitalisation. We calculated the incremental cost-effectiveness ratio (ICER) of targeted RSV prophylaxis vs. no prophylaxis per quality-adjusted life year (QALYs) using a societal perspective, including medical and parental costs and effects. Costs and health outcomes were modelled in a decision tree analysis with sensitivity analyses. Targeted RSV prophylaxis in infants with a first-year RSV hospitalisation risk of > 10% resulted in a QALY gain of 0.02 (0.931 vs. 0.929) per patient against additional cost of €472 compared to no prophylaxis (ICER €214,748/QALY). The ICER falls below a threshold of €80,000 per QALY when RSV prophylaxis cost would be lowered from €928 (baseline) to €406 per unit. At a unit cost of €97, RSV prophylaxis would be cost saving.

*Conclusions*: Targeted RSV prophylaxis is not cost-effective in reducing RSV burden of disease in moderately preterm infants, but it can become cost-effective if lower priced biosimilar palivizumab or a vaccine would be available.

**Electronic supplementary material:**

The online version of this article (10.1007/s00431-017-3046-1) contains supplementary material, which is available to authorized users.

**Table:** 

**What is known:** • *RSV infection has a high burden of disease in preterm infants leading to hospitalisations and recurrent wheezing during the first year of life.* • *Due to high costs , the cost-efffectiveness of RSV prophylaxis is the subject of debate, a targeted prophylaxis strategy could positively impact the cost-effectiveness analyses in a time of health care budget constraints.*
**What is new:** • *Our results show that targeted RSV prophylaxis is not cost-effective, but it can become cost-effective if a biosimilar palivizumab becomes available at 40% of the cost of current RSV prophylaxis.*

## Introduction

Respiratory syncytial virus (RSV) bronchiolitis is a major cause of infant morbidity in both high income and low- and middle-income countries and is associated with a large burden of disease and high costs [15, 20, 30, 37]. A systematic review estimated the global incidence among children < 1 year of age at 19.19 per 1000 infants per year and a threefold higher rate for preterm infants [38]. Each year, about 28,000 infants require medical care for RSV bronchiolitis in the Netherlands [21, 28], of which approximately 2000 require hospitalisation with costs of €2000–€4000 per patient [9, 23, 33]. In moderately preterm infants born at 32–35 weeks gestational age (WGA), we recently reported that about 9% of infants require mechanical ventilation at a paediatric intensive care unit (PICU) [25].

Children most at risk for severe disease are prematurely born infants either with or without chronic lung disease (CLD) and children with congenital heart disease (CHD) [3]. RSV prevention is possible with a RSV-specific biological, palivizumab. RSV prophylaxis has shown to be effective in preventing RSV infection in preterm infants < 35 WGA [8, 39]. We showed in our randomised clinical trial that RSV infection has a causal relation with recurrent wheeze during the first year of life in such infants [8]. Although the burden of disease is considerable, RSV-associated mortality in healthy term infants is probably low, but published estimates vary between 0 and 8% [15, 30, 31, 34, 36, 38].

Meijboom estimated the total annual cost to society in the Netherlands due to RSV to be €7.7 million if no vaccination is undertaken [28]. Due to high costs, the cost-effectiveness of RSV prophylaxis is the subject of vigorous debate [1, 2, 22, 32]. Several systematic reviews of the cost-effectiveness of palivizumab conclude that results vary considerably and are sensitive to poor-quality input values, especially the RSV-associated mortality rate [2, 5, 43]. The current RSV prophylaxis program with palivizumab for preterm infants born before 32 WGA and infants with CLD or CHD includes 2994 users in the Netherlands and the total annual cost was €14.0 million for 2015 [16].

Following the publication of the MAKI trial (no acronym), we raised the issue to perform a formal cost-effectiveness analysis based on trial data and including impact and prevention of recurrent wheeze [6, 10]. Our trial provided us with a population of preterm infants 33–35 WGA randomly assigned to RSV prophylaxis or placebo with associated detailed follow-up of RSV burden of disease and health care consumption. We further integrated incidence data of the large RISK birth cohort study in preterm infants 32–35 WGA designed to develop a validated prediction rule for RSV hospitalisation risk. To approximate real-time health care choices, we included in our base case analysis the risk prediction at birth to determine the impact of targeted RSV prophylaxis in preterms with a > 10% hospitalisation risk [25]. Integration of decision rules and targeted treatment programmes in recent cost-effectiveness analyses to define cost-effective or even cost-saving strategies in a time of health care budget constraints is an accepted approach but remains rare [12, 18, 35, 42]. Because our trial spanned three subsequent RSV seasons (2008–2011) and the RISK birth cohort study spanned seven consecutive RSV seasons (2008–2014), our data reflects the heterogeneity of RSV seasonality. The aim of this study is to determine the cost-effectiveness of targeted RSV prophylaxis in late preterm infants 32–35 WGA using a prospectively validated prediction rule compared to standard care, i.e. no prophylaxis.

## Methods

### Model

**Fig. 1:**
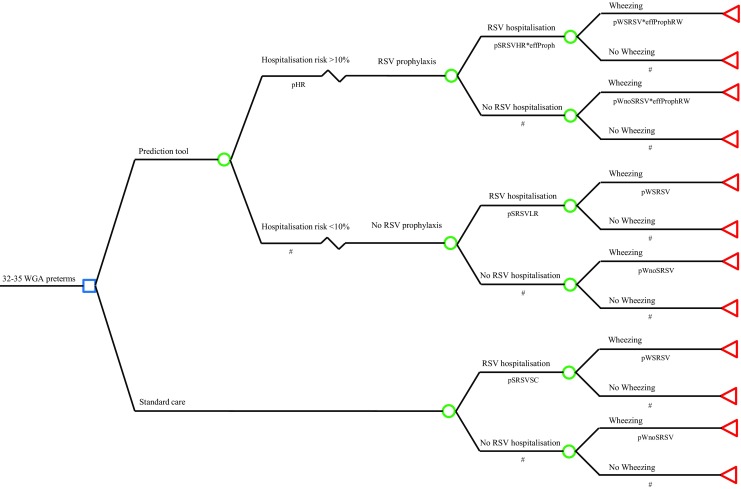
Decision tree analysis for targeted RSV prophylaxis in moderate preterm infants

This cost-effectiveness study was performed based on the MAKI randomised, double blind, placebo-controlled, multicenter trial and the RISK birth cohort study, reported in more detail elsewhere [8, 25]. A cost-utility analyses (CUA) was conducted to assess the economic benefit of targeted RSV prophylaxis with humanised monoclonal antibody palivizumab compared to no prophylaxis in moderately preterm infants born at 32–35 WGA for reducing the burden of RSV infection. The outcome of the CUA was incremental costs per quality-adjusted life year (QALY) gained. This analysis reflects the extra costs of preventive treatment, i.e. RSV prophylaxis, minus the prevented health care cost in relation to the prevented decrease in health care burden due to RSV-related illness, i.e. QALY gain by prevention of RSV hospitalisation and subsequent wheezing. The analysis was performed from a societal perspective, which includes not only medical costs but also societal costs as made by parents. For the base case analysis, for which input values were not yet varied, a time horizon of 1 year was used which matches the time horizon of the MAKI trial. We choose to build a decision tree to avoid substantial, and potentially unreliable, extrapolation of trial data and implemented a validated prediction rule to target RSV prophylaxis at infants with increased risk of severe RSV disease (Fig. 1) [7, 25]. No discounting, a technique to correct cost and outcome inputs derived from different time periods, was necessary due to the 1-year horizon. The decision tree model was built using TreeAge Pro (2017, TreeAge Software Inc., Williamstown, MA, USA).

### Participants and randomization

In short, in the MAKI trail, 429 moderately preterm infants (gestational age, 33 to 35 weeks) were recruited from paediatric departments of one university hospital and 15 regional hospitals in the Netherlands. Eligible infants were randomly assigned in a 1:1 ratio to receive either monthly intramuscular palivizumab injections or placebo during the winter season [8].

In short, in the RISK study of a multicenter prospective birth cohort in 41 hospitals in the Netherlands, we validated a prediction rule (area under the receiver operating curve 0.72 (95% CI 0.65–0.78)) in 4088 moderately preterm infants to identify a high-risk group with a hospitalisation risk ≥ 10% in the first year of life which is comparable to the hospitalisation risk in preterm infants, < 32 WGA and other high-risk groups [7, 25]. Risk factors (e.g. day care attendance, presence of siblings, birth period) were assessed at birth among healthy preterm infants 32–35 WGA. All hospitalisations for respiratory tract infection were screened for laboratory-proven RSV infection.

### Probabilities and clinical data

Probabilities on disease incidence were derived from the MAKI trial and the RISK birth cohort study (Table 1). The MAKI trial was designed and powered to determine wheezing incidence; therefore, incidence of recurrent wheeze was derived from this source. Because the incidence of RSV hospitalisations was low in the MAKI trial, we derived probabilities and duration of RSV admission and PICU admission from the RISK study. We included mortality estimates that were derived from the Dutch RSV Mortality Study, a study on RSV-associated mortality. This study provided Dutch RSV mortality estimates derived from hospital PICU administration and the Dutch Central Bureau of Statistics (CBS) (Supplemental information).

**Table 1:** Model inputs: morbidity probabilities used in base case and sensitivity analyses

Model input	Base case value	SA range for one-way sensitivity analyses^a^	Distribution	Source
Probability				
Prediction rule				
High risk (> 10% RSV hospitalisation risk)	0.112	0.08–0.14	*β* (SD 0.01)	Korsten et al.
RSV prophylaxis group				
Recurrent wheezing, no RSV hospitalisation^b^	0.19	0.15–0.24	*β* (SD 0.02)	Blanken et al.
Recurrent wheezing, RSV hospitalisation^b^	0.55	0.41–0.68	*β* (SD 0.05)	Blanken
RSV hospitalisation, given high risk	0.126	0.095–0.158	*β* (SD 0.01)	Korsten
PICU, given hospitalisation^c^	0.088	0.07–0.11	*β* (SD 0.01)	Korsten
Mortality, given PICU admission^c^	0.01	0.008–0.013	*β* (SD 0.001)	Supplement
Placebo group				
Recurrent wheezing, no RSV hospitalisation	0.19	0.15–0.24	*β* (SD 0.02)	Blanken
Recurrent wheezing, RSV hospitalisation	0.55	0.41–0.68	*β* (SD 0.05)	Blanken
RSV hospitalisation, given low risk	0.034	0.026–0.043	*β* (SD 0.005)	Korsten
PICU, given hospitalisation	0.088	0.07–0.11	*β* (SD 0.01)	Korsten
Standard care				
Recurrent wheezing, no RSV hospitalisation	0.19	0.15–0.24	*β* (SD 0.02)	Blanken
Recurrent wheezing, RSV hospitalisation	0.55	0.41–0.68	*β* (SD 0.05)	Blanken
RSV hospitalisation	0.044	0.033–0.055	*β* (SD 0.005)	Korsten
PICU, given hospitalisation	0.088	0.07–0.11	*β* (SD 0.01)	Korsten
Utility (positive)/disutility (negative)				
No RSV hospitalisation, baseline	0.95	0.71–1.00	Gamma (SD 0.1)	Greenough et al.
RSV hospitalisation	− 0.07	− 0.05– -0.09	Gamma (SD 0.01)	Greenough
PICU admission^§^	− 0.15	− 0.17– -0.28	Gamma (SD 0.02)	Jones et al.
Wheezing, QALY reduction	− 0.08	− 0.06– -0.1	Gamma (SD 0.01)	RIVM
Prophylaxis effectiveness				
Reduction of RSV hospitalisation	0.82	0.62–1.03	β (SD 0.08)	Blanken
Reduction of recurrent wheezing	0.47	0.35–0.59	β (SD 0.05)	Blanken

*SA range* sensitivity analysis range, *SD* standard deviation

^a^Univariate sensitivity analysis ranges were derived by increasing and decreasing baseline values by 25%

^b^Recurrent wheezing following RSV GP visit in the RSV prophylaxis group was assumed equal to recurrent wheezing following RSV GP visit in the placebo group because the trial data suggested an inconsistent probability of 1.0 following RSV GP visit in the RSV prophylaxis group (*n* = 2)

^c^Potential utility loss and costs due to PICU admission and mortality were included in all RSV hospitalisation based on the probability of PICU admission and mortality following RSV hospitalisation

### Follow-up

In the MAKI trial, parents recorded airway symptoms, doctor visits, hospitalisations and the use of airway medication in a daily log until their infant was 1 year of age. General practitioners (GPs) recorded number of GP visits and number of prescriptions of short-acting beta agonist as relief medication (first-choice test treatment Dutch College of GPs) [4]. In this model, we included recurrent wheeze in the first year of life. Recurrent wheeze was defined as three or more episodes of wheezing during the first year of life. The number of hospitalisations for laboratory-proven RSV infection was assessed during the first year of life in both the MAKI trial and the RISK study.

### Measurement of effectiveness

The efficacy of RSV prophylaxis with palivizumab in reducing hospital admission in infants born at 32–35 weeks gestational age was set at 82% (95% CI 18–157%) reduction as retrieved from two randomised clinical trials [8, 39]. Additionally, the MAKI trial provided the efficacy of RSV prophylaxis in reducing recurrent wheeze which was set a 47% reduction [8].

### High-risk group identification

For the use of targeted RSV prophylaxis, we considered 11% of the palivizumab group as high risk, with a cut-off of a > 10% hospitalisation risk, following the proportion of the RSV prediction rule paper [25]. The MAKI trial data did not permit us to do individualised prediction because of missing baseline data for the prediction rule and the low percentage of hospitalisations [8].

### Cost estimates

We valued the use of health care resources for both treatment groups in the MAKI trial with Dutch reference prices and calculated total costs from the total quantity of health care resources consumed and the unit cost of those resources [19]. Costs of medication were obtained from the Dutch Formulary, including a pharmacist fee for each subscription. Use of bronchodilators (short-acting beta agonist, first choice salbutamol/albuterol) was calculated for a trial course of 2 weeks, followed by symptom relief treatment based on reported symptoms in the diary, according to national asthma guidelines for this age group [4]. Over the counter drugs were not measured in the trial and not included in this model because of a lack of reliable data in this population. Used health care resources did not include administration cost for RSV prophylaxis as this is a free of charge service as part of palivizumab reimbursement in the Netherlands. In case of PICU admission, ambulance transfer was taken into account because PICU admissions in the Netherlands generally occur after a transfer from a secondary to a tertiary care hospital. Parental transportation costs were calculated based on the estimate of 189 travelled kilometres (km) per admission and reference costs of €0.9 per km [19, 29]. Other costs included productivity losses by caretakers as a result of caregiving to children suffering from RSV infections. It has been estimated that on average two parental workdays are lost as the result of a RSV-related hospitalisation [29].

### Health outcomes

In the model, utilities were defined for all health states, and using the health state durations (i.e. modelled at 1 year), QALYs were calculated for each strategy to determine the QALY gains for the targeted RSV prophylaxis strategy compared to no prophylaxis. One study by Greenough et al. provides utilities for RSV health states for preterm children with a RSV hospitalisation. In this study, the quality of life in children, aged 2–4 years, with a history of preterm birth and RSV hospitalisation were compared with that of a control group of preterm children without a history of RSV hospitalisation [17]. The median Health Utilities Index (HUI 2) multi-attribute utility function was 0.88 in children with a confirmed RSV infection and a history of chronic lung disease, as compared to 0.95 in the control group. For quality of life loss following a PICU admission, we included the HUI 2 score of 0.73 from a study of 1455 children, mean age 4 years, who were followed up until 6 months after discharge [24]. To prevent double counting, we assumed that this decrease in quality of life due to a PICU admission is not additive to the QALY decrease due to a RSV admission because this PICU admission would also include an initial hospital admission. QALY decreases due to recurrent wheezing were not separately assessed in these studies therefore we based the quality of life decrease on the best estimate as derived from QALY decrease for asthma of 0.08 based on a Dutch national reference study [13].

### Sensitivity analyses

It is important to evaluate to uncertainty of the input values used in a cost-effectiveness analysis. To account for this univariate and probabilistic sensitivity, analyses were performed to explore the impact of parameter uncertainty. Transition probabilities were inserted as beta distributions and utility decrements as gamma distributions [11]. Cost-related parameters were inserted as fixed values when prices were fixed (i.e. GP visits). To measure the impact of the used baseline cost and outcome variables, these were varied by increasing and decreasing baseline inputs by 25% to account for a wide range of uncertainty. Univariate sensitivity analyses on all key input variables were conducted increasing and decreasing each input variable while keeping other variables constant to identify the critical parameters driving results. Results of one-way sensitivity analyses were depicted in a tornado diagram. In addition, probabilistic sensitivity analysis was performed to evaluate the uncertainty of the ICER taking into account uncertainty across all variables simultaneously. In this analysis, the base case estimate and a distribution (e.g. normal, beta, gamma, log-normal, fixed) were specified (Table 2). With Monte Carlo sampling, 5000 samples were drawn from these distributions and used as input for the model, so the model was run 5000 times to evaluate the difference in account with the difference in input. Each iteration produced values for incremental costs, incremental benefits and ICERs. From the 5000 simulations, the probability that the intervention is cost-effective (net monetary benefit > 0, given a willingness to pay €80,000) was deduced and the 95% CI.

**Table 2:** Unit prices of resources used for preterm infants during 1-year trial follow-up

Resource	Unit cost (€)	Source
Intervention costs		
Specialist hourly fee	104	Hakkaart et al, 2015
Palivizumab, per unit^a^	928.60	GIP databank
Pharmacist fee	6	Farmacotherapeutisch Kompas
Direct medical costs		
GP contact, unit	33	Hakkaart
Hospital admission paediatrics, per day	627	Hakkaart
Ambulance transfer, urgent^b^	613	Hakkaart
PICU admission, per day	2015	Hakkaart
Wheezing GP contact	28	Hakkaart
SABA episode, including Babyhaler	21.5	Medicijnkosten.nl
Indirect medical costs		
Parental costs		
Transportation (per km)	0.19	Hakkaart
Workdays lost	278	Hakkaart

All unit costs are based on 2015 prices. Based on fixed reference prices not included in sensitivity analyses

^a^Price year 2015

^b^Additive to PICU admission cost

### Threshold analyses

A threshold analysis of lower prophylaxis prices on the ICER was also analysed, to determine the maximum cost of RSV prophylaxis for which the targeted RSV strategy would have an incremental cost-effectiveness ratio less than the informal threshold of €80,000/QALY [41]. All analyses were performed with TreeAge Pro and SPSS version 20 (IBM SPSS Statistics, Chicago, IL).

## Results

### Participants

The MAKI trial consisted of 429 moderately preterm infants included at birth. Of these, 214 infants were randomly assigned to receive palivizumab and 215 infants were assigned to receive placebo. The two groups were well balanced regarding inclusion year, gestational age and birth month and had similar baseline characteristics as described previously (Supplementary Table) [8]. The RISK study consisted of 4088 moderately preterm infants included at birth with a follow-up period of 1 year.

### Costs, health outcomes and cost-effectiveness

Unit prices and mean use of resources per infant during the 1-year trial follow-up were evaluated (Table 1, 2 and 3). During the 1-year follow-up, the mean total RSV prophylaxis costs per patient were €4717 for the RSV prevention group and €0 for the placebo group. A separate analysis of trial data only produced an ICER of > €1,000,000/QALY when targeted prophylaxis was not considered. The analysis of health outcomes showed that targeted RSV prophylaxis resulted in 0.0022 QALYs gained (0.931 vs. 0.929) at an additional cost of €472 (€758 vs. €286) per patient compared to no prophylaxis. Targeted RSV prevention with palivizumab for moderately preterm infants vs. no prophylaxis in the base case produced an ICER of €214,748 per QALY gained.

**Table 3:** Mean use of resources

Resource	Palivizumab (*n* = 214)	Placebo (*n* = 215)
Intervention costs		
Specialist fee		
Palivizumab prescription (hour)	0.08^a^	0
Palivizumab units^b^	5.08	0
Pharmacist fee total	43.5	0
Direct medical costs		
Hospital admission, RSV proven (SD)^c^	5.8 days (4.8)	5.8 days (4.8)
Ambulance transfer, given PICU admission	1	1
PICU admission (SD)^d^	8.1 days (8.0)	8.1 days (8.0)
Recurrent wheezing GP contact^e^ (SD)	2.5 (2.2)	5.3 (5.8)
Episodes with SABA prescription^f^ (SD)	0.12 (0.6)	0.21 (0.5)
Indirect medical costs		
Parental costs given hospital admission		
Transportation (km)^g^	189	189
Work days lost^g^	2	2

Values are means

^a^Duration for prescription based on personal communication

^d^Based on Dutch national GIP (The Drug Information System of National Health Care Institute) databank data of actual yearly palivizumab use (SABA: short-acting beta agonists)

^c^Based on the RSV-positive admissions in the RISK study (*n* = 181, hospital laboratory proven, Korsten et al.); the number of RSV-positive admissions in the MAKI trial: RSV prophylaxis (*n* = 2, mean duration 5.3 days), placebo (*n* = 11, mean duration 6.6 days)

^d^Based on the RISK study PICU admission duration (Korsten et al.), there were no PICU admission in the RSV prophylaxis group and 1 PICU admission in the placebo group, duration 10.75 days

^e^GP reported

^f^GP or parent reported, corrected for double counting

^g^Not recorded in the MAKI trial, derived from Miedema et al.

### Sensitivity analyses

**Fig. 2:**
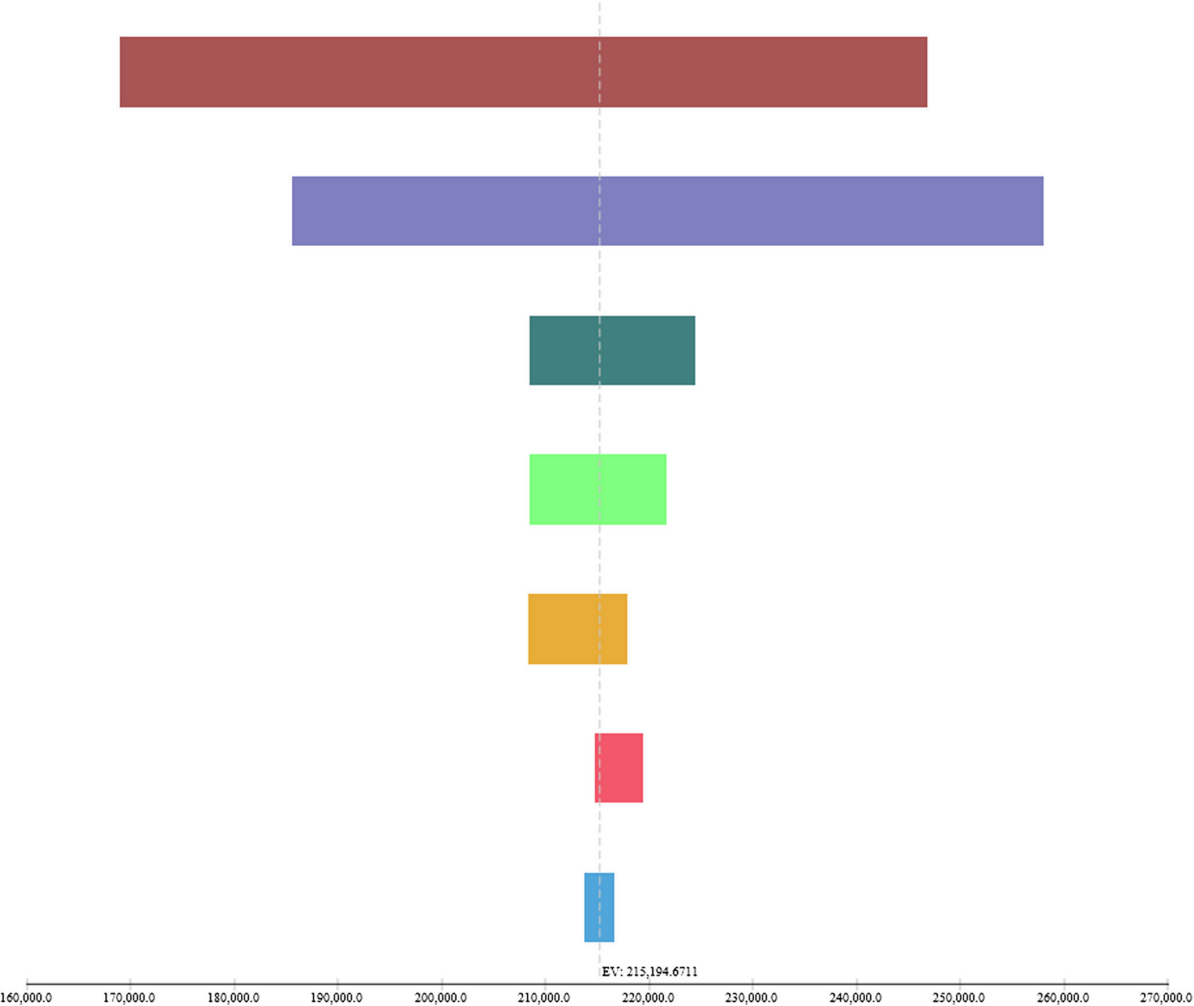
One-way sensitivity analyses, tornado diagram. Values are ICER €/QALY with tornado bars representing the effect of univariate sensitivity analyses. Variables were selected based on level of impact (from top to bottom): high-risk probability of the prediction rule, RSV prophylaxis effectiveness in preventing RSV hospitalisations, the RSV hospitalisation incidence in the high-risk population, the hospital admission duration, the probability of PICU admission following RSV hospitalisation, the probability of mortality following PICU admission and the PICU admission duration

**Fig. 3:**
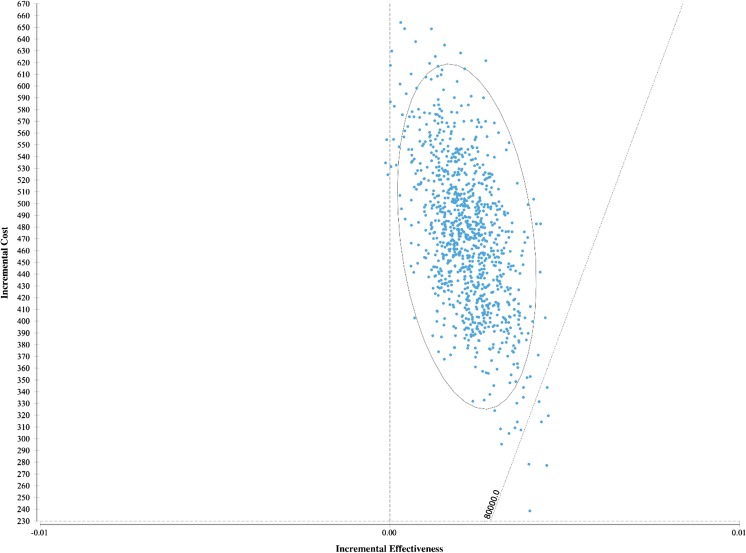
Incremental cost-effectiveness scatterplot on a cost-effectiveness plane showing the statistical uncertainty through 5000 bootstrapped samples. Results of probabilistic sensitivity analysis with per infant incremental cost-effectiveness in a scatterplot for targeted RSV prophylaxis vs. standard care (no RSV prophylaxis) in moderately preterm infants 32–35 weeks gestational age. The reference line represents willingness to pay threshold of €80,000/QALY

**Fig. 4:**
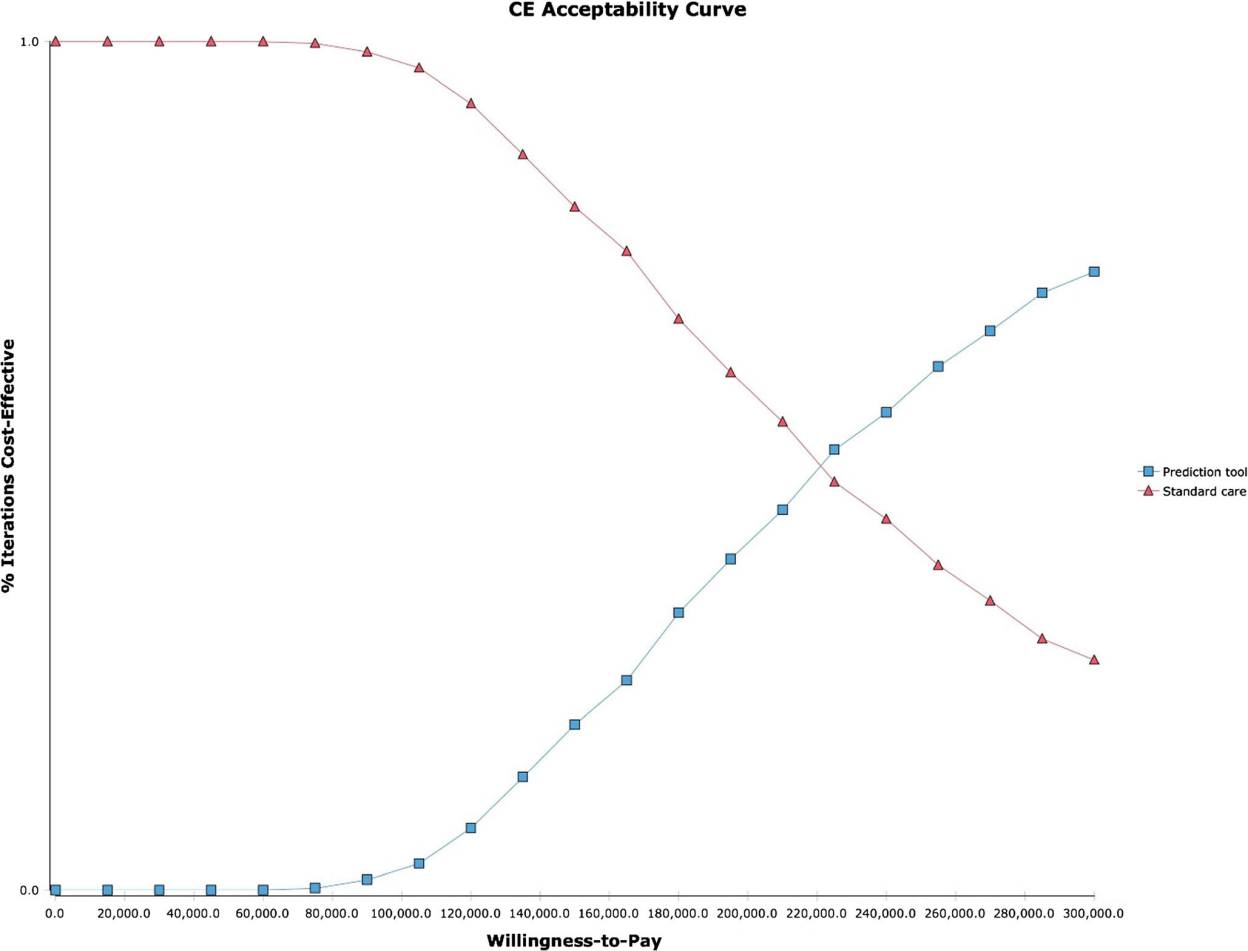
Cost-effectiveness acceptability curve at different willingness to pay levels for RSV prophylaxis based on 5000 iterations. Results of probabilistic sensitivity analysis with per infant incremental cost-effectiveness in a cost-effectiveness acceptability curve of targeted RSV prophylaxis (blue line) vs. standard care (no RSV prophylaxis, red line) in moderately preterm infants 32–35 weeks gestational age

Figure 2 shows that the ICER was most sensitive to the discriminatory power of the prediction rule (range €168,996–€246,852/QALY) and the RSV prophylaxis effectiveness (range €185,637–€258,055/QALY). The effect of PICU incidence and the effect of mortality following PICU were limited (range €208,327–€217,955/QALY and range €214,834–€219,427/QALY). Furthermore, the effects of the cost of RSV hospitalisation and PICU admission following RSV hospitalisation were limited (range €208,519–€221,674/QALY and range €213,769–216,620/QALY) (Fig. 2). The probabilistic sensitivity analysis showed that the probability of cost-effectiveness is 0.5% considering a threshold of €80,000 (Fig. 3). The cost-effectiveness acceptability curve shows the performance of targeted RSV prophylaxis compared to standard care at different willingness to pay levels (Fig. 4).

### Threshold analysis

In the scenario analysis to evaluate the effect of lower priced RSV prophylaxis, lowering the price of the treatment with RSV prophylaxis from €929 to €406 per unit (€2062 per infant per year) assuming future market introduction of a biosimilar anti-RSV humanised monoclonal antibody yields a favourable ICER below the informal threshold of €80,000 per QALY. At a unit cost < €97 (€493 per infant per year), RSV prophylaxis would become cost saving in this high-risk population.

## Discussion

Our results show that targeted RSV prophylaxis results in an incremental cost-effectiveness ratio of €214,748 per QALY gained and therefore is not a cost-effective strategy to prevent severe RSV infection and wheeze in the first year of life. Even with targeted RSV prophylaxis for only 10% of moderately preterm infants with an estimated risk of > 10% for RSV hospitalisation, the costs are still well above the informal Dutch cost-effectiveness threshold €80,000 per QALY gained. We are the first to present targeted cost-effectiveness of RSV prophylaxis compared to no prophylaxis in moderately preterm children based on prospective trial data and a large birth cohort study. The use of RSV prophylaxis in this high-risk population results in a small increase in QALYs against high additional costs. One-way and probabilistic sensitivity analyses showed the robustness of our results and impact of individual parameters on the outcome.

Subsequent threshold analyses showed that the current available RSV prophylaxis, palivizumab, would need a 60% price cut for an acceptable cost-effectiveness level at a threshold of €80,000 per QALY. A price cut of > 90% would result in a cost-saving strategy. Currently, a palivizumab biosimilar is under investigation at the Utrecht Centre for Affordable Biotherapeutics but the progress is unknown [40]. Taken together, our study helps to understand acceptable pricing for future RSV preventive interventions, in particular palivizumab biosimilars for otherwise healthy late preterm infants.

The major strength of our study is that it is the first cost-effectiveness study of RSV prophylaxis in this population based on data from a randomised placebo-controlled trial and a large birth cohort study, which enabled us to include the most reliable baseline probabilities and include all relevant evidence as deemed appropriate by Briggs et al. [11]. Some limitations should also be discussed. First, we did not assess all use of resources in our trial. Therefore, we used published data from the Dutch costing manual and published data for resource use. For indirect cost made by parents, we included estimates from a Dutch paper better representing our population rather than estimates from a more comprehensive analysis in moderately preterm infants [26, 29]. Second, due to the choice for a short time horizon in line with trial follow-up, the impact of mortality following severe RSV infection is limited. However, the Dutch RSV Mortality Study described that RSV-related mortality in otherwise healthy preterm infants is minimal. Third, the utility estimates were derived from the literature because with the quality of life estimates from our trial we could not determine utility scores for RSV infection. In our trial, we took the TNO-AZL Preschool Children Quality of Life (TAPQOL) questionnaire every 3 months. However, TAPQOL does not report utilities [14]. As a consequence, deriving QALY decreases due to RSV admission or PICU admission from different sources could lead to an effect underestimation because we assumed that not all QALY decreases were additive, for example in the case of PICU admission. This will likely not have influenced the results of our study, since the number of PICU admissions is low.

The RSV treatments that are currently in development include 10 vaccines and 11 therapeutic agents in active clinical trials [27]. Maternal vaccination is especially relevant for infants below 6 months of age, as these infants are at high risk for severe disease but are unlikely to benefit from active immunisation. It is our understanding that, even with the introduction of a maternal or infant vaccine, the use of anti-RSV monoclonal antibodies could still be necessary to protect preterm infants below the age of 3–6 months. The use of a maternal or infant vaccine is highly dependent on level of efficacy and the age at first vaccination and could implicate a time horizon for monoclonal antibody protection before vaccination is possible and effective. Our model could be easily adapted to consider a combination of RSV prophylaxis with monoclonal antibody and new RSV vaccines.

## Conclusion

Targeted RSV prophylaxis is not yet cost-effective in reducing RSV burden of disease in moderately preterm infants with incremental costs per QALY ratio far exceeding applied threshold values. Our results show that targeted RSV prophylaxis could become cost-effective if lower priced biosimilar palivizumab or a vaccine becomes available.

## Electronic supplementary material

ESM 1 (DOCX 27 kb)

## Abbreviations

CHD: Congenital heart disease
CI: Confidence interval
CLD: Chronic lung disease
GP: General practitioner
ICER: Incremental cost-effectiveness ratio
PICU: Paediatric intensive care unit
QALY: Quality-adjusted life year
RSV: Respiratory syncytial virus
WGA: Weeks gestational age
