# Supplementary material for: Cost-effectiveness of rule-based immunoprophylaxis against respiratory syncytial virus infections in preterm infants
Source: Eur J Pediatr. 2017 Nov 22;177(1):133–44. doi: 10.1007/s00431-017-3046-1 (PMC5748402; doi:10.1007/s00431-017-3046-1)
Supplement: Supplementary file 1 — (DOCX 27 kb) [file 431_2017_3046_MOESM1_ESM.docx]

Supplementary material

Manuscript

Cost-effectiveness of rule-based immunoprophylaxis against respiratory syncytial virus infections in preterm infants

Supplementary Online content

Sections

1. Baseline characteristics MAKI trial
2. Dutch mortality study

Supplementary Table 1. Baseline characteristics MAKI trial

|  | Palivizumab  (n=214) | Placebo  (n=215) |
| --- | --- | --- |
| Male (%)** | 125 (58%) | 94 (44%) |
| Birth Weight – gram (95%CI) | 2294 (1363-3325) | 2289 (1385-3358) |
| Gestational Age – weeks (95%CI) | 34+3 (32+2-35+6) | 34+3 (32+3-35+6) |
| Multiple birth (%) | 38 (19%) | 36 (18%) |
| Type of feeding (%) |  |  |
| Breastfeeding and formula | 90 (44%) | 107 (53%) |
| Breastfeeding | 59 (29%) | 49 (24%) |
| Formula | 54 (27%) | 46 (23%) |
| Maternal smoking during pregnancy (%) | 32 (15%) | 34 (16%) |
| Parental smoking |  |  |
| Mother (%) | 33 (15%) | 36 (17%) |
| Father (%) | 57(27%) | 62 (29%) |
| Siblings (%) | 82 (44%) | 85 (45%) |
| Age mother (median (range) | 31 (19-48) | 32 (18-44) |
| Age father (median (range) | 34 (21-55) | 35 (22-52) |
| Atopy Mother (%) | 85 (40%) | 72 (34%) |
| Physician diagnosis Asthma | 22 (11%) | 24 (12%) |
| Physician diagnosis Hay fever | 48 (24%) | 45 (23%) |
| Physician diagnosis Eczema | 48 (24%) | 30 (15%) |
| Atopy Father (%) | 73 (34%) | 80 (37%) |
| Physician diagnosis Asthma | 27 (14%) | 21 (11%) |
| Physician diagnosis Hay fever | 44 (22%) | 52 (26%) |
| Physician diagnosis Eczema | 29 (15%) | 27 (14%) |
| Household pets (%) | 97 (48%) | 98 (49%) |
| Daycare attendance (%)  Sibling attending daycare (%) | 103 (48%)  75 (37%) | 113 (53%)  79 (40%) |
| Doses received (median (range) | 4 (1-5) | 4 (2-5) |

Data are represented as mean (95% confidence interval) or number (percentage); **: p<0.01. These data were previously published as an online supplement to the original MAKI trial paper (Blanken et al. NEJM)

Dutch mortality study

For this study we combined information from all Dutch pediatric intensive care units (PICUs), and death certificates of the Dutch Central Bureau for Statistics (CBS). The aim of this study is to determine the mortality rate of RSV (respiratory syncytial virus) LRTI (lower respiratory tract infection) in children in the Netherlands and study the influence of comorbidity in RSV mortality. We investigated the number of deaths for Dutch children from birth to 3 years of age in 2003-2008, using mortality data of Dutch PICUs, the Dutch CBS and the Dutch Forensic Institute (NFI). In case of acknowledged RSV mortality, the following information was determined: sex, age, weeks of gestational age, birth weight, positive RSV infection, RSV prophylaxis, all pre-existent comorbidity, comorbidity coinciding with RSV infection, diagnosis on PICU admission, age of death, cause of death and length of stay (LOS). Data of children with the diagnosis bronchiolitis were obtained from all Dutch PICUs: Academic Medical Center University Amsterdam, VU Medical Center Amsterdam, University Medical Center Groningen, Leiden University Medical Center, Maastricht University Medical Center, University Medical Center Nijmegen St. Radboud, Erasmus Medical Center and University Medical Center Utrecht. After permission for data extraction from the Dutch PICUs, the required information was obtained from the national Pediatric Intensive Care Evaluation registry (PICE registry). Individual PICU patient files of deceased children were investigated in case of RSV infection. The PICE registry is a database of all children admitted to a PICU in the Netherlands since 2003. This registry is filled in by trained pediatric intensivists. The purpose of this registry is to give more insight in diseases, treatment and prognosis, to compare the different departments leading to a better health management and to evaluate changes in policy. The PICE registry provided a database with children aged 0-3 years admitted to a PICU with bronchiolitis over the period 2003-2008 and a database of all deceased children aged 0-3 years who were admitted to a PICU with a bronchiolitis. Of one PICU, the Wilhelmina’s Children Hospital in Utrecht, all deceased children on the PICU among 2003-2008 were retrospectively investigated for RSV infections by checking individual patient files. At the same time cooperation with the Dutch Central Bureau for Statistics (CBS) was sought. The CBS collects all death certificates in the Netherlands. The cause of death is filled in on a death certificate by a physician and describes whether the cause of death was natural or unnatural. On the form is also the opportunity to describe all relevant comorbidities. The CBS gave us information about the total mortality of children aged 0-3 years for the period 2003-2008 and specified cases where RSV was mentioned as cause of death or relevant comorbidity. When looking at the period 2003-2008, there were 1099 children with a bronchiolitis admitted on a PICU in the Netherlands. Of those children, 16 did not survive their PICU admission. Four children were excluded because they did not have a RSV bronchiolitis. Viruses that were found were Influenza A virus, adenovirus, parechovirus and one child had a picornavirus, rhinovirus and cytomegalovirus. Two children were found by checking the patient files of the deceased children in the Wilhelmina’s Children Hospital. Of the 14 children there were 7 boys and 7 girls. Most children died in the months December through February. The mean age of death was 11 months. The mean length of stay on the PICU was 18 days. All children had severe comorbidity. Comorbidities mentioned were haemophagocytic lymphohistiocytosis (HLH), Bordetella Pertussis infection, congenital heart diseases, familiar erythrophagocytic lymphohistiocytosis (FEL), metabolism disorders (e.g. Pompe disease), epilepsy, protein S deficiency, thrombosis, Wolcott Rallison syndrome, acute respiratory distress syndrome (ARDS), pulmonary hypertension, Miller Dieker syndrome, chromosomal abnormalities, congenital immune deficiency, encephalopathy, fungal, viral and bacterial infections, severe combined immune deficiency (SCID) and myopathy. The annual number of mortality cases of children admitted to a PICU with a RSV infection was 2.3 children per year over this six-year period. This means that 1 in 82.000 children or 0.00122% of the annually born children will die with a RSV infection. Annually, 183 children are admitted to a PICU with a bronchiolitis. Of those children, approximately 1 in 80 children or 1.26% will die with a RSV infection. In the last six years, annually a mean of 188.874 children were born alive in the Netherlands. In the period of 2003-2008 the total amount of mortality cases of children aged 0-3 years was 5.863 deaths, registered in the CBS for the Netherlands. Seven of these deaths were primarily attributable to RSV (0.12%) of whom four (57.1%) had severe comorbidity. Comorbidities that are mentioned in this group are Bordetella Pertussis infection, glycogen storage disease, Werdnig Hoffman disease, unspecified spinal muscle atrophy, degenerative disease of basal ganglia, unspecified chronic respiratory disorder, spina bifida not specified, Arnold Chiari syndrome and congenital heart anomaly not specified. Seven children died with RSV as secondary cause of death (0.12%) of whom 100% had severe comorbidity. Comorbidities that are mentioned in this group are metabolism disorder not specified, mitochondrial myopathy, atrioventricular septal defect, chondrodysplasia punctata and Edwards syndrome. In total 14 children, of whom 11 (78.6%) were less than one year of age, died with a positive RSV infection. This means that annually 2.3 children will die with a positive RSV infection which means an annually mortality rate of 0.00122% of all living born children. Eleven of these children had severe comorbidity, while three children had no comorbidity. Based on CBS data, in children with comorbidity the mortality rate is 0.00095% as compared to 0.00026% in healthy children. Because there is special interest in preterm children we attempted to estimated RSV-related mortality in this sub-population, but gestational age data were not provided by the CBS. If we would assume that 7% of Dutch infants are born prematurely and all deaths had occurred in preterm infants, which is believed to be a strong overestimation, the mortality would be 0.017%. In the period 2003-2008 there were 14 children who died on a PICU in the Netherlands with a positive RSV infection of whom 100% had severe comorbidity. According to the CBS, there were also 14 children who died with a positive RSV infection, but only 78.6% had severe comorbidity. When looking for the hidden mortality by investigating sudden infant death by the NFI, no positive RSV infections were found. Using the available data, the estimated mortality rate due to RSV in all annually born children is 0.00122%. For preterm children without comorbidity the estimated mortality is probably negligible, but an upper limit of mortality rate for this population was calculated to be 0.017% (≈1 per 6.000 preterm children). In conclusion, a raw estimate of RSV-associated mortality appears to be low.
